# Influence of Powder Milling and Annealing Parameters on the Formation of Cubic Li_7_La_3_Zr_2_O_12_ Compound

**DOI:** 10.3390/ma14247633

**Published:** 2021-12-11

**Authors:** Dariusz Oleszak, Mirosława Pawlyta, Tomasz Pikula

**Affiliations:** 1 Faculty of Materials Science and Engineering, Warsaw University of Technology, 02-507 Warsaw, Poland; 2 Institute of Engineering Materials and Biomaterials, Silesian University of Technology, 44-100 Gliwice, Poland; miroslawa.pawlyta@polsl.pl; 3 Faculty of Electrical Engineering and Computer Science, Lublin University of Technology, 20-618 Lublin, Poland; t.pikula@pollub.pl

**Keywords:** lithium-ion batteries, solid-state electrolyte, solid-state reaction, mechanical milling, nanopowders, XRD, SEM, TEM

## Abstract

Li-ion batteries are widely used as energy storage devices due to their excellent electrochemical performance. The cubic Li_7_La_3_Zr_2_O_12_ (c-LLZO) compound is regarded as a promising candidate as a solid-state electrolyte for lithium-ion batteries due to its high bulk Li-ion conductivity, excellent thermal performance, and chemical stability. The standard manufacturing procedure involves the high-temperature and lengthy annealing of powders. However, the formation of the tetragonal modification of LLZO and other undesired side phases results in the deterioration of electrochemical properties. The mechanical milling of precursor powders can enhance the powders’ reactivity and can result in an easier formation of c-LLZO. The aim of this work was to study the influence of selected milling and annealing parameters on c-LLZO compound formation. The starting powders of La(OH)_3,_ Li_2_CO_3_, and ZrO_2_ were subjected to milling in various ball mills, under different milling conditions. The powders were then annealed at various temperatures for different lengths of times. These studies showed that the phase transformation processes of the powders were not very sensitive to the milling parameters. On the other hand, the final phase composition and microstructure strongly depended on heat treatment conditions. Low temperature annealing (750 °C) for 3 h produced 90% of c-LLZO in the powder structure.

## 1. Introduction

From an energy storage technology point of view, rechargeable Li-ion batteries are the most advanced energy storage devices. Li-ion batteries possess the highest energy densities (the capability to store energy per unit weight or volume) among all rechargeable batteries, which is important regarding its applications in large electrical power storage systems such as electrical vehicles [1,2,3]. Additionally, these batteries typically have relatively low self-discharge rates compared to Ni-metal hydride or Ni-Cd batteries. Moreover, Li-ion batteries have attracted attention because of its safety issues; it should be stressed that most liquid electrolytes are extremely flammable [4]. However, their application is still limited mainly to portable electronic devices such as mobile phones, cameras, or notebook computers. Within the last decade, Li-ion-conducting oxides with garnet-related structures have garnered attention as solid-state electrolytes [3,5,6]. Particularly, the Li_7_La_3_Zr_2_O_12_ (LLZO) compound is regarded to be a promising candidate as the solid-state electrolyte for lithium-ion batteries due to its high bulk Li-ion conductivity, excellent thermal performance, and chemical stability [5]. Although LLZO may be a key material for battery application, there are many issues related to its fabrication. Various processing routes can be applied for solid-state electrolytes: sol-gel synthesis techniques have been attempted with varying degrees of effectiveness [7]; laser annealing is another important fabrication technique [8]; spark plasma sintering has also been used, however, with a porous microstructure being reported [9], along with calcination and cold compaction of the powders [10]; finally, hot press sintering has also been developed [11]. However, the most frequently used technique is a standard procedure that involves the mixing of appropriate powders and their high temperature annealing (1300–1500 °C) for a lengthy period (24–36 h) [7,11,12]. These processes are time- and energy-consuming. Additionally, the loss of lithium [5] and the formation of harmful, undesired side phases after annealing/sintering are frequently observed. Moreover, the appearance of tetragonal modification (t-LLZO) in the structure results in the deterioration of the conductivity of the material [5]. Typical bulk Li-ion conductivity of c-LLZO at room temperature is 5 × 10^−4^ S cm^−1^, while for tetragonal modification, the value is lower by two orders of magnitude [6]. The formation of cubic modification (c-LLZO) is more beneficial due to its superior electrochemical performance due to fast Li-ion diffusion and the positions and populations of the Li sites [6]. Cubic modification of LLZO can also be stabilized by the addition of some elements [5,13,14,15,16,17]. Therefore, the application of mechanical milling (MM) of precursor powders prior to their annealing/sintering can enhance the powders’ reactivity and can result in an easier formation of c-LLZO. Several works have been devoted to the determination of the influence of milling parameters on the phase composition and structure of the final product [18,19,20,21,22,23,24,25]. These parameters can include the stoichiometry of the starting powder mixture, doping, type of mill (impact or shear mode), ball-to-powder weight ratio (BPR), material of vials and balls, size of balls, milling time, process control agent (PCA), and the atmosphere of milling. However, not all of them have been studied in detail. This work was focused on the development of the chosen technological parameters related to milling and annealing and their influence on the phase composition of the final ceramic, while the electrochemical properties of the synthesized powders were not studied. Thus, the aim of this work was to study the influence of selected milling and annealing parameters on the possibility of c-LLZO compound formation.

## 2. Materials and Methods

For milling processes, the powders of ZrO_2_ (purity 99.9%, Tosoh, Tokyo, Japan), La(OH)_3_ (99.9%, Aldrich, Darmstadt, Germany), and Li_2_CO_3_ (99.8%, Aldrich, Darmstadt, Germany) were used. The size of all powders was submicrometric. The powders were mixed in a molar ratio of 3.5:3:2, corresponding to the stoichiometry of Li_7_La_3_Zr_2_O_12_. The milling processes were performed in a Fritsch P5 (Fritsch GmbH, Idar-Oberstein, Germany) planetary ball mill at 250 rotations per minute for various lengths of milling times, as well as in a SPEX8000D mixer (SPEX, Metuchen, NJ, USA). The two types of mills used show different ways in which the energy is transferred to the milled powders. In an SPEX mill, the back-and-forth shaking motion is combined with lateral movements of the ends of the vial. Because of the amplitude and speed, the force of the ball’s impact is great. On the other hand, in a Fritsch planetary mill, both impact and friction effects are present due to ball movement and, as a consequence, in comparison to an SPEX mill, Fritsch can be considered a lower energy mill [26]. In both cases, an alumina container and alumina balls with an 8 mm diameter were used. The ball-to-powder weight ratio was 45:1. The milling processes were performed in air and no process control agent was added. After milling, the powders were subjected to annealing in a laboratory furnace in air at 750 and 950 °C for 2 and 3 h. The phase composition of the powders subjected to milling and annealing was examined by X-ray diffraction using a Rigaku Mini Flex II diffractometer (Rigaku, Tokyo, Japan) equipped with Cu -K*α* radiation (*λ* = 0.15418 nm). Detailed analysis of the obtained diffractograms was performed using the High Score Plus computer program (Malvern Panalytical Ltd., Malvern, UK). The Rietveld method was applied for the refinement of the crystalline structure [27]. The following parameters were refined during the Rietveld procedure: scale factor, lattice parameters, Caglioti parameters (U, V, and W), relative coordinates of atoms in unit cell, and isotropic thermal displacement parameters. As the pseudo-Voight function was used for peak shape reproduction, the peak shape parameters, γ, were also refined.

SEM observations of powder size and morphology were performed using a Hitachi S3500 unit (Hitachi, Tokyo, Japan). For TEM studies, specimens were prepared by dispersing a small amount of the sample in methanol and putting a droplet of the suspension on a microscope copper grid covered with carbon. TEM investigations were undertaken with a field-emission transmission electron microscope (S/TEM Titan 80-300 FEI, Eindhoven, The Netherlands) with a super twin lens operated at 300 kV and equipped with an annular dark-field detector. The chemical composition was determined in the same apparatus using energy dispersive spectroscopy EDS (EDAX, Tilburg, The Netherlands).

## 3. Results and Discussion

**Figure 1:**
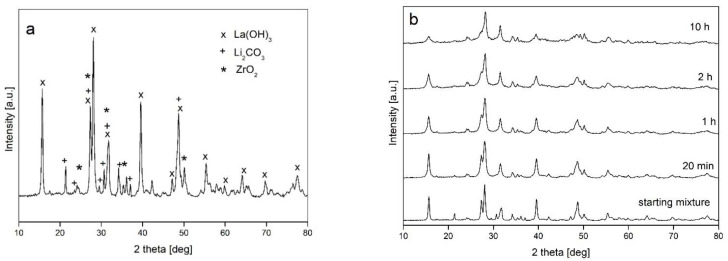
XRD patterns registered for: (**a**) starting powder mixture; (**b**) samples subjected to mechanical milling in a Fritsch P5 planetary ball mill with increasing milling times.

Figure 1a shows the X-ray pattern for the starting mixture of powders. The lines corresponding to three precursor compounds are visible. The sequence of XRD patterns related to samples subjected to milling in a Fritsch P5 planetary ball mill with increasing processing time is shown in Figure 1b. It can be seen that there were no phase transformations during milling. Due to structural factors, the pattern is dominated by lines corresponding to lanthanum hydroxide. As a result of mechanical milling, the diffraction lines of all compounds significantly broadened and their intensity decreased. The changes of peak height and width with milling time are typical for powders subjected to mechanical milling and can be attributed to microstructural refinement and the generation of crystal imperfections upon mechanical milling. These changes were observed practically up to 1 h of milling, and prolonged processing up to 10 h did not influence the registered XRD pattern. Therefore, for further experiments, a processing time of 1 h was selected.

**Figure 2:**
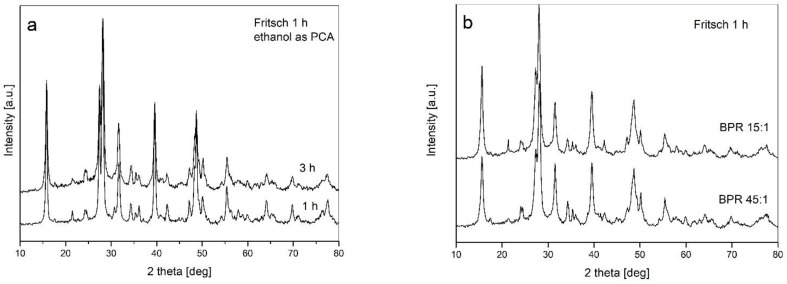
XRD patterns registered for powders subjected to mechanical milling in a Fritsch P5 planetary ball mill: (**a**) with ethanol addition; (**b**) with various BPRs applied.

For studying the influence of PCA addition on the milling process and the structural transformations, 10 wt.% of ethanol was chosen. The milling experiment was performed in a Fritsch P5 planetary ball mill. Figure 2a shows the registered diffraction patterns for powders milled with ethanol for 1 and 3 h. It can be seen that both patterns match. The intensities and widths of the recorded diffraction lines are identical. The prolongation of milling had no influence on the process; moreover, the presented XRD patterns are very similar to the one recorded for 1 h of processing without ethanol addition condition. Additionally, the diffraction lines in this case exhibited even smaller broadening than for milling without PCA. Thus, the addition of ethanol as the PCA in this case is not a beneficial factor from a structural refinement point of view.

The influence of ball-to-powder weight ratio (BPR) is presented in Figure 2b. It can be seen that even a significant increase in milling energy due to an increase in BPR from 15:1 to 45:1 did not result in visible changes in the observed X-ray patterns. Therefore, both an addition of ethanol as he PCA as well as changes in BPR had no influence on the observed transformations, and thus testified for a weak sensitivity of the examined powder mixture to the milling parameters applied.

**Figure 3:**
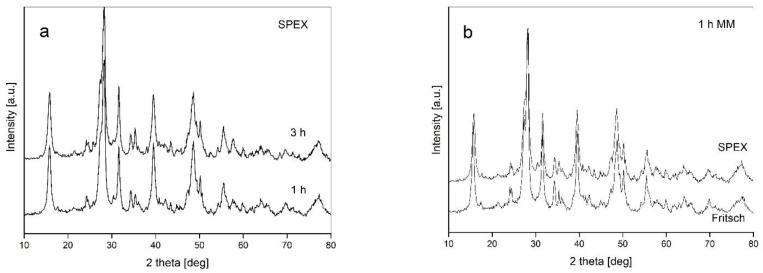
XRD patterns recorded for: (**a**) the samples milled in an SPEX mixer for various times; (**b**) the samples synthesized in various mills with the same milling time (1 h) applied.

The next experiment was performed with the use of an SPEX mixer device. This type of mill, contrary to the planetary Fritsch mill, exhibits a rather high energy impact type of interaction between the balls and powders. However, the XRD patterns presented in Figure 3a for two significantly different milling times show that there is no influence of milling time on phase transformations. The patterns after 1 and 3 h are practically identical and reveal the broadened diffraction lines corresponding to the starting compounds. Simultaneously, the X-ray patterns recorded after the same milling time (1 h) using two different mills show no differences (Figure 3b). This result shows that the application of different mills providing various contributions regarding impact and friction modes of milling has minor influence on the processing of powders.

**Figure 4:**
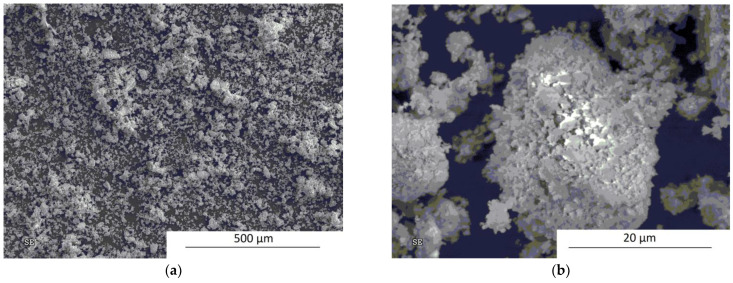
SEM micrographs showing the morphology and size of powder particles after 1 h of mechanical milling in a Fritsch planetary ball mill: (**a**) low and (**b**) high magnification.

The SEM micrographs revealing the particle size and morphology after 1 h of processing in a Fritsch P5 planetary ball mill are presented in Figure 4a,b. The powders show a strong tendency toward agglomeration. The submicrometric particle size is clearly visible.

**Figure 5:**
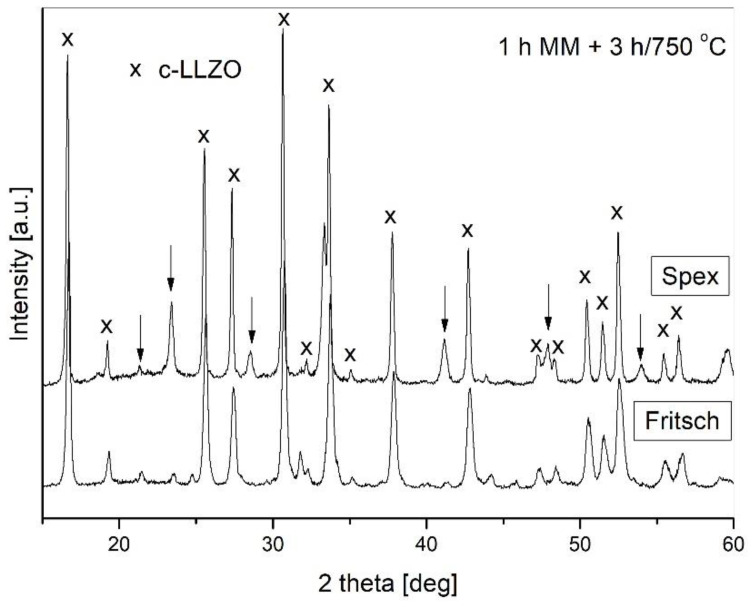
X-ray patterns for samples subjected to milling and subsequent annealing.

The obtained results confirmed that the mechanochemical processes performed did not lead to the direct formation of LLZO, as only structural refinement was observed. Therefore, the next set of experiments was related to powder annealing. The powders were annealed in air at temperatures ranging from 750 to 950 °C for various lengths of times. Figure 5 shows the XRD patterns for the powders subjected to milling in both types of mills for 1 h and subsequently annealed at 750 °C for 3 h. These studies revealed that the applied parameters of annealing were sufficient for the formation of c-LLZO. In both cases c-LLZO was the dominant phase. The diffraction lines corresponding to other phases are marked by an arrow. It can be seen that for the SPEX mixer, the contribution of these side phases is slightly higher than that from the Fritsch mill.

**Figure 6:**
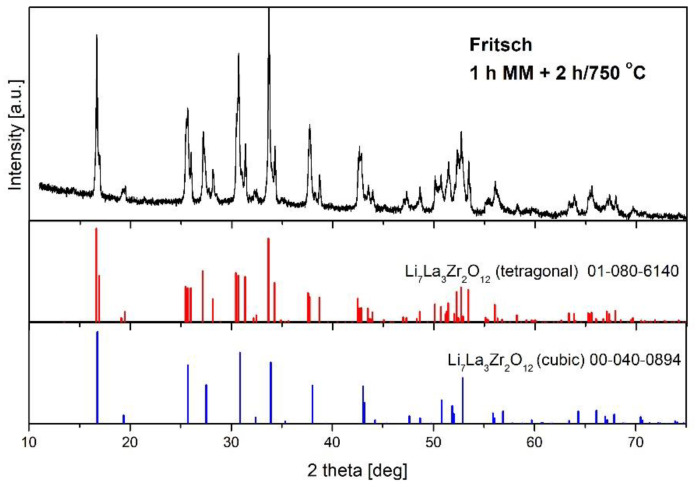
X-ray pattern for samples subjected to milling in a Fritsch P5 planetary mill and annealed at 750 °C for 2 h, and the angle positions of diffraction lines corresponding to both modifications of LLZO.

In order to calculate the content of c-LLZO and to identify remaining phases, Rietveld analysis was performed. Rietveld analysis was especially useful for distinguishing between both modifications of LLZO (cubic and tetragonal), as their diffraction lines overlap, as shown in Figure 6.

**Figure 7:**
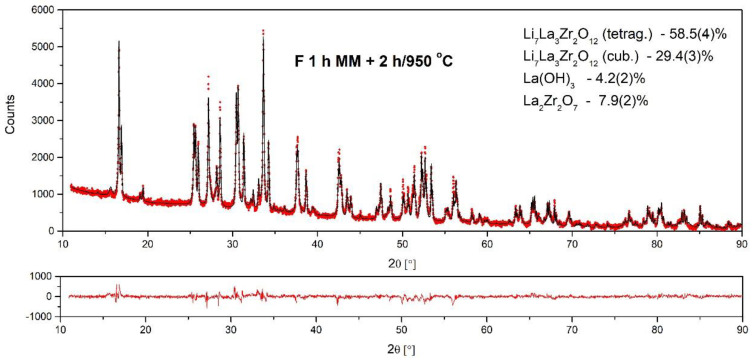
Rietveld analysis for the sample annealed in air at 950 °C for 2 h.

Figure 7 shows an example of the results of the Rietveld analysis on the sample synthesized in a Fritsch ball mill for 1 h and subsequently annealed at 950 °C for 2 h. The lattice parameter of c-LLZO was calculated as a = 13.025 Å, which agrees well with data reported in the literature [28]. The values of the fitting parameters usually used for the estimation of the fitting quality (χ^2^ = 3, R_exp_ = 5.9, R_prof_ = 8.5, and R_w prof_ = 10.3) support the good quality of the obtained results. The details of the performed analysis are presented in Table 1.

Depending on the milling and annealing conditions, different contributions of the desired c-LLZO compound were detected in the structure of the studied samples. By analyzing the influence of annealing time, it was found that for each heat treatment temperature, a prolongation of annealing resulted in an increase in c-LLZO content. On the other hand, a lower annealing temperature was more beneficial from a c-LLZO formation point of view. Other compounds found in the studied samples were divided into two types: unreacted starting precursors or new compounds formed during powder annealing. There may have been different reasons for their formation. Hydrated La_2_O_2_ tends to react with the carbon dioxide in air. Thus, the amount of lanthanum in the final ceramic could have caused multiphasic composition after milling and heat-treatment procedures. The escape of lithium was also reported [5]. The presence of the compound containing Al (LaAlO_3_) could be explained by the use of an alumina container and balls for powder processing. A summary of the Rietveld analysis of several of the studied samples is presented in Table 2.

**Figure 8:**
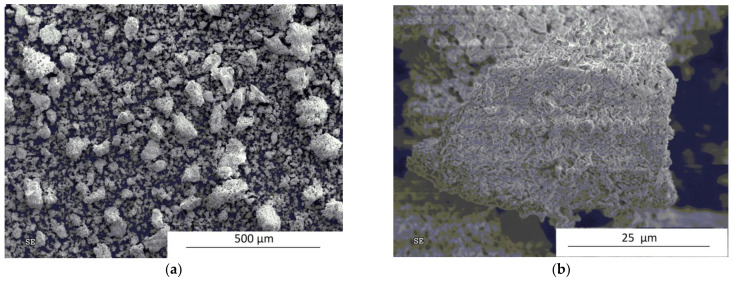
SEM micrographs showing the powders after 1 h of mechanical milling in a Fritsch planetary ball mill and annealing at 750 °C for 3 h: (**a**) low and (**b**) high magnification.

SEM studies were performed for the annealed powders as well. The micrographs revealing the powders after 1 h of processing in a Fritsch P5 planetary ball mill and subsequent annealed at 750 °C for 3 h are presented in Figure 8. Comparing the powders milled only, it can be seen that after annealing, the powders underwent at least partial sintering (Figure 8b).

**Figure 9:**
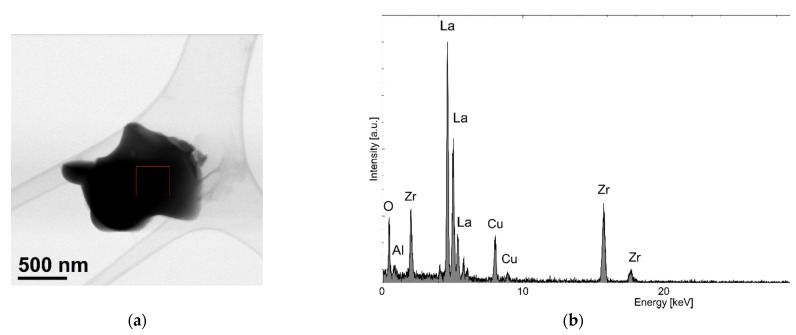
TEM micrograph showing the morphology and size of powder particles after 1 h of mechanical milling in a Fritsch planetary ball mill (**a**) and EDS analysis results (**b**).

For structural studies of the powders after milling for 1 h in a Fritsch P5 planetary ball mill, TEM observations were performed. Figure 9a reveals a single powder particle with a size of approximately one micrometer and of irregular shape. The EDS analysis (Figure 9b) confirmed the presence of La, Zr, and O. The presence of Cu may have been an artefact, while Al came from the milling media (alumina).

## 4. Conclusions

This work focused on studying the influence of mechanical milling and annealing parameters on the formation of single-phase, cubic modification of the Li_7_La_3_Zr_2_O_12_ compound. Mechanical milling processes were performed in two different ball mills (Fritsch and SPEX). The starting mixture of submicrometric precursor powders consisted of La(OH)_3_, Li_2_CO_3_, and ZrO_2_ mixed in appropriate proportions. After milling the powders were subjected to annealing in air at various temperatures for different lengths of times.

The following conclusions can be drawn from the performed experiments:

- The mechanical milling processes had no induced phase transformations of the starting powder mixture, and a broadening of the diffraction lines was observed only due to structural refinement and the generation of crystal imperfections;
- The type of ball mill used (with different dominant modes of milling—impact and friction) has no influence on the processing of powders
- Changes to other milling parameters (ball-to-powder weight ratio BPR, addition of ethanol, prolongation of milling up to 10 h) do not introduce qualitative changes in the powder structure;
- The formation of c-LLZO was possible only after powder annealing, and the amount of the desired cubic modification of LLZO significantly depended on the annealing parameters. It was found that annealing at 750 °C resulted in a higher amount of c-LLZO than processing at 950 °C, and that the prolongation of annealing time for upward of 3 h was beneficial.

## Figures and Tables

**Table 1:** Rietveld analysis for the sample annealed in air at 950 °C for 2 h.

Compound	Weight Content (%)	Cryst. Structure	Space Group	Lattice Parameters (Å)
Li_7_La_3_Zr_2_O_12_	29.4 (3)	cubic	IA–3d	a = 13.025 (1)
Li_7_La_3_Zr_2_O_12_	58.5 (4)	tetragonal	I41/acd	a = 13.1189 (8), c = 12.6698 (5)
La_2_Zr_2_O_7_	7.9 (2)	cubic	Fd-3m	a = 10.8226 (8)
La(OH)_3_	4.2 (2)	hexagonal	P63/m	a = 6.5346 (5), c = 3.8579 (5)

**Table 2:** The weight content of c-LLZO in the structure of the studied samples and the list of other phases detected, depending on milling and annealing parameters.

Milling and Annealing Parameters	c-LLZO Content (wt.%) ±2 (%)	Other Phases Detected
Fritsch 1h MM + 750 °C/2h	48	t-LLZO Li_2_CO_3_ ZrO_2_ La_2_Zr_2_O_7_ LaAlO_3_ La(OH)_3_ La_2_O_2_CO_3_ La_2_O_3_ LaOOH
Fritsch 1 h MM + 750 °C/3 h	90	
Fritsch 1 h MM + 950 °C/2 h	30	
Fritsch 1 h MM + 950 °C/3h	60	
SPEX 1 h MM + 750 °C/3 h	80	

## Data Availability

The data presented in this study are available on request from the corresponding author.
